# Physiological and biochemical effect of neem and other Meliaceae plants secondary metabolites against Lepidopteran insects

**DOI:** 10.3389/fphys.2013.00359

**Published:** 2013-12-20

**Authors:** Sengottayan Senthil-Nathan

**Affiliations:** Division of Biopesticides and Environmental Toxicology, Sri Paramakalyani Centre for Excellence in Environmental Sciences, Manonmaniam Sundaranar UniversityTirunelveli, India

**Keywords:** Meliaceae, secondary metabolites, insect, food consumption, enzyme activity

## Abstract

This review described the physiological and biochemical effects of various secondary metabolites from Meliaceae against major Lepidopteran insect pest including, Noctuidae and Pyralidae. The biochemical effect of major Meliaceae secondary metabolites were discussed more in this review. Several enzymes based on food materials have critical roles in nutritional indices (food utilization) of the insect pest population. Several research work has been referred and the effect of Meliaceae secondary metabolites on feeding parameters of insects by demonstrating food consumption, approximate digestibility of consumed food, efficiency of converting the ingested food to body substance, efficiency of converting digested food to body substance and consumption index was reviewed in detail. Further how the digestive enzymes including a-Amylases, α and β-glucosidases (EC 3.2.1.1), lipases (EC 3.1.1) Proteases, serine, cysteine, and aspartic proteinases affected by the Meliaceae secondary metabolites was reviewed. Further effect of Meliaceae secondary metabolites on detoxifying enzymes have been found to react against botanical insecticides including general esterases (EST), glutathione S-transferase (GST) and phosphatases was reviewed. Alkaline phosphatase (ALP, E.C.3.1.3.1) and acid phosphatase (ACP, E.C.3.1.3.2) are hydrolytic enzymes, which hydrolyze phosphomonoesters under alkaline or acid conditions, respectively. These enzymes were affected by the secondary metabolites treatment. The detailed mechanism of action was further explained in this review. Acethylcholine esterase (AChE) is a key enzyme that terminates nerve impulses by catalyzing the hydrolysis of neurotransmitter, acetylcholine, in the nervous system of various organisms. How the AChE activity was altered by the Meliaceae secondary metabolites reviewed in detail.

## Introduction

Crop protection all over the world relies heavily on the use of synthetic pesticides. In the past, synthetic pesticides have played a major role in crop protection programes and have immensely benefited mankind. The discovery and use of DDT in 1940 and then BHC and subsequent development of the chlorinated cyclodienes marked a major advance in the field of crop protection. These chemicals have made great contributions to plant protection but have also raised a number of ecological and medical problems (Varma and Dubey, 1999). Nevertheless, their indiscriminate use has resulted in the development of resistance by pests (insects, weeds, etc), resurgence and outbreak of new pests, toxicity to non-target organisms and hazardous effects on the environment endangering the sustainability of ecosystems (Jeyasankar and Jesudasan, 2005). It has been estimated that hardly 0.1% of the agrochemicals used in crop protection reach the target pest leaving the remaining 99.9% to enter the environment to cause hazards to non-target organisms including humans (Pimentel and Levitan, 1986).

It has been described that more than 2.5 million tons of pesticides are used in agricultural crops protection for every year and the global damage caused by synthetic insecticides reaches more than $100 billion annually (USEPA, 2011). The reason behind this amount of cost is the high toxicity and residual properties of pesticides in soil, water, air and crops that affect human and domestic animal health (Carson, 1951). Hence search for the eco-friendly, biodegradable pesticides for management of pest insects have been encouraged to be essential for last five decades.

The ideal insecticide should control target pests adequately and should be target-specific (able to kill the pest insect but not other insects or animals), rapidly degradable, and low in toxicity to humans and other mammals. Two classes of insecticides that exhibit some of these characteristics are the botanical insecticides and the insecticidal soaps. Botanical insecticides, sometimes referred to as “botanicals,” are naturally occurring insecticides have been derived from plants. Insecticidal soaps are soaps that have been selected and formulated for their insecticidal action (Weinzierl and Henn, 1991).

Botanical insecticides have more advantages than synthetic one. The advantages of botanical pesticides mainly depending upon their quick degradation and lack of persistence and bioaccumulation in the eco system, which have been key problems in chemical pesticide use.

Several experiment with botanical pesticides, shows they are degraded in the environment in hours or days. Further literature has clearly shown that use of plant natural products provides low risk when compare with chemical insecticides. The availability and diversity of the secondary metabolites in botanical extracts is renewable source. Also multiple analogs of one compound, is known to increase the efficiency of phytochemcial through synergism, reduce the rate of metabolism of the compounds and prevent the pest resurgence/pesticide resistance (Ascher, 1993; Senthil-Nathan and Kalaivani, 2005, 2006; Ntalli and Menkissoglu-Spiroudi, 2011). Plant community is the most efficient source for natural pesticide. It synthesizes numerous products, many of which have been shown to effect on insect and other harmful organism. Some are highly toxic to a wide range of organisms, including both vertebrates and invertebrates. But majority of plant derived compounds are affecting insects and are comparatively harmless to vertebrates. Such compounds are toxic causing mortality or reduced growth of pest insects. Phytochemcial modes-of-action are more complicated. Most of them are affecting insect performance by repelling an insect and feeding deterrence or oviposition deterrence.

The reviews on plant secondary metabolites/phytochemicals are extensive and have been reviewed by several authors (Arnason et al., 1987; Champagne et al., 1989; Rosenthal and Berenbaum, 1992; Harborne, 1993; Tan and Luo, 2011). But secondary metabolites on insect biochemical mode of action including enzyme activity are still obscure. This review has been mainly attempt to emphasis the biochemical mode of action of *Meliaceae* secondary metabolites against Lepidopteran insect pests.

## Biological activities of meliaceae plants against Lepidopteran insects

The Meliaceae plant family has been given much attention due to its chemical characters called “limonoid” (Connolly, 1983). Meliaceae are distributed in tropical and subtropical regions throughout the world with 50 genera and more than 1400 species (Tan and Luo, 2011). The term limonoids was originated from limonin, the first tetranortriterpenoid acquired from bitter principles of citrus fruits (Devakumar and Sukhdev, 1993; Roy and Saraf, 2006). Current research has pointed out that limonoids are highly oxygenated, modified terpenoids with wide range biological activities especially action against the insects. Not only insecticidal activity it has antibacterial, antifungal, antimalarial, anticancer, antiviral and other clinical activities on humans (Roy and Saraf, 2006). Some reviews related to limonoids from Meliaceae have been presented since 1966. It is noteworthy that some reviews emphasize the well-known azadirachtin (Kraus et al., 1985) and aspects of its chemistry, synthesis (Ley et al., 1993; Sundaram, 1996; Ley, 2005; Devakumar and Kumar, 2008) and bioactivities including antifeedant activity, insecticidal activity and insect-growth-regulating activity (Schmutterer, 1990; Mordue and Blackwell, 1993; Simmonds and Blaney, 1996) as well as its environmental behavior (Sundaram, 1996) and its physiological behavior properties (Mordue and Blackwell, 1993; Mordue, 2004) (Table 1). In addition, the toxicity characteristics of azadirachtin and the mechanisms of its insecticidal action were also reviewed (Champagne et al., 1989; Rembold, 1989). The Indian neem tree (*Azadirachta indica* A. Juss), one of the important limonoid producing plants from Meliaceae family, has long been recognized as a source of environment-friendly biopesticide. Several constitutions of its leaves and seeds show marked insect control potential and due to their relative selectivity, neem products can be recommended for many Integrated Pest Management (IPM) programs (Schmutterer, 1990).

**Table 1:** **Biochemical effect of Meliaceae plants secondary metabolites against the Lepidopteran insects**.

**Plant name**	**Action against the insect**	**Mode of action**	**Authors**
*Aglaia cordata*	*Spodoptera frugiperda*	GI	Mikolajczak and Reed, 1987
*A. oilo*	*Peridroma saucia*	GI	Satasook et al., 1994
*A. odorata*	*Peridroma saucia*	GI	Satasook et al., 1994
*A. maiae*	*Peridroma saucia*	GI	Satasook et al., 1994
*A. iloilo*	*Peridroma saucia*	GI	Satasook et al., 1994
*A. odorata*	*Peridroma saucia*	GI	Satasook et al., 1994
*A. ohgophy*	*Peridroma saucia*	GI	Satasook et al., 1994
*A. odorata*	*Peridroma saucia*	GI	Satasook et al., 1994
Azadirachta excels	*Crocidolomia binotalis*	GI	Teik Ng et al., 2003
*Azadirachta indica*	*Achoea janata*	FD	Ramachandran et al., 1989
	*Agrotis ipsilon*	FD	George and Potter, 2008
	*Helicoverpa armigera*	FD	Katti et al., 1992
	*Heliothis virescens*	FD	Lee et al., 1988
	*Mythimna separate*	FD	Schmutterer et al., 1983
	*Cnaphalocrocis medinalis*	FD	Schmutterer et al., 1983
	*Ephestia kuhniella*	FD	Rembold et al., 1980
	*Peridroma saucia*	FD	Isman et al., 1990
	*Peridroma plorans*	FD	Champagne et al., 1989
	*Ostrinia nubilalis*	FD	Arnason et al., 1985
	*Ascotis selenaria*	FD	Meisner et al., 1976
	*Achaea janata*	FD	Chari and Muralidharan, 1985
	*Trichoplusia ni*	GI	Prabhaker et al., 1986
	*Spodoptera exigua*	GI	Prabhaker et al., 1986
	*Spodoptera frugiperda*	FD	Kubo and Klocke, 1982a,b
	*Spodoptera littoralis*	FD	Meisner et al., 1981
	*Spodoptera litura*	FD	Koul, 1987
	*Earias fabia*	OI	Pathak and Krishna, 1986
	*Earia insulana*	GI	Meisner et al., 1978
	*Earias vittella*	OI	Sojitra and Patel, 1992
	*Pectinophora gossypiella*	GI	Salem, 1991
	*Haritalodes (*also: *Sylepta) derogata*	GI	Cobbinah and Osei-Owusu, 1988
	*Sesamia calamistis*	GI, OI	Bruce et al., 2004
	*Eldana saccharina*	GI, OI	Bruce et al., 2004
	*Plutella xyllostella*	GI	Verkerk and Wright, 1993
	*Plodia interpunctella*	GI, EI	Rharrabe et al., 2008
	*Choristoneura fumiferana*	FD	Thomas et al., 1992
	*C. rosaceana*	EI	Smirle et al., 1996
	*Macalla thyrsisalis*	FD	Howard, 1990
	*Pieris brassicae*	FD	Arpaia and Loon, 1993
	*Manduca sexta*	GI	Haasler, 1984
	*Mamestra brassicae*	OI	Shimizu, 1988
*Carapa guianensis*	*Spodoptera frugiperda*	FD	Sarria et al., 2011
*Cabralea canjerana*	*Spodoptera frugiperda*	FD	Sarria et al., 2011
*Cedrela odorata*	*Hypsipyla grandella*	FD	Soto et al., 2007
*Cedrela salvadorensis*	*Ostrinia nubilalis*	GI	Jimenez et al., 1997a,b
	*Spodoptera frugiperda*	GI	Céspedes et al., 2000
*Cipadessa fruticosa*	*Spodoptera frugiperda*	GI	Matos et al., 2009
*Cedrela dugessi*	*Spodoptera frugiperda*	GI	Céspedes et al., 2000
*Dysoxylum malabaricum*	*Cnaphalocrocis medinalis*	EI	Senthil-Nathan et al., 2007
*Dysoxylum beddomei*	*Cnaphalocrocis medinalis*	NPI	Senthil-Nathan et al., 2007
*Entandrophragma* spp.	*Oslrinia nubilalis*	GI	Arnason et al., 1987
*Entandrophragma candolei*	*Helicoverpa armigera*	FD,NPI	Koul et al., 2003
*Khaya ivorensis*	*Agrotis segetum*	GI	Vanucci et al., 1992
*Khaya senegalensis*	*Spodoptera littoralis*	GI	Nakatani et al., 2004
*Lanium domesticum*	*Spodoptera litura*	GI	Leatemia and Isman, 2004
*Melia azedarach*	*Cnaphalcrocis medinalis*	FD,EI	Senthil-Nathan, 2006
	*Hyblaea puera*	FD,EI	Senthil-Nathan and Sehoon, 2006
	*Pieris brassicae*	GI	Atwal and Pajni, 1964
	*Agrotis ipsilon*	EI	Schmidt et al., 1997, 1998
	*Spodoptera littoralis*	EI,OI	Schmidt et al., 1997
	*Spodoptera eridania*	GI	Nakatani, 1999
	*Earias vittella*	OI	Gajmer et al., 2002
	*Thaumetopoea pityocampa*	GI	Breuer and Devkota, 1990
	*Sesamia nonagrioides*	GI	Juan et al., 2000
	*Plutella xylostella*	GI	Dilawari et al., 1994
	*Spodoptera frugiperda*	GI	Mikolajczak et al., 1989
	*Busseola fusca*	GI	Gebre-Amlak and Azerefegne, 1999
	*Tuta absoluta*	GI	Brunherotto and Vendramim, 2001
	*Thaumatopoea pityocampa*	GI	Breuer and Devkota, 1990
	*T. processionea*	FD	Breuer and Loof, 1998
	*Phthorimaea operculella*	GI	Kroschel and Koch, 1996
	*Sesamia nonagrioides*		
*Melia dubia*	*Spodoptera litura*	GI,FD	Koul et al., 2000
	*Helicoverpa armigera*	GI,FD	Koul et al., 2000
*Melia volkensii*	*Spodoptera frugiperda*	FD	Rajab et al., 1988
	*Trichoplusia ni*	GI	Isman, 2005
*Melia toosendan*	*Trichoplusia ni*	GI	Isman, 2005
	*Peridroma saucia*	GI	Chen et al., 1995
	*Spodoptera litura*	GI,EI	Feng et al., 1995
*Munronia henryi*	*Pieris brassicae* L.	FD	Qi et al., 2003
*Sandoricum koetjape*	*Spodoptera frugiperda*	GI	Powell et al., 1991
	*Spodoptera litura*	FD,GI	Leatemia and Isman, 2004
*Swietenia humilis*	*Ostrinia nubilalis*	GI	Jimenez et al., 1997a,b
*Teucrium tomentosum*	*Plutella xylostella*	FD	Krishna-Kumari et al., 2003
	*Spodoptera litura*	FD	Krishna-Kumari et al., 2003
*T. connaroides*	*Peridroma saucia*	GI	Xie et al., 1994
	*S. litura*	GI	Xie et al., 1994
*T. glabra*	*Peridroma saucia*	GI	Xie et al., 1994
	*S. litura*	GI	Xie et al., 1994
*T. hirta*	*Peridroma saucia*	GI	Xie et al., 1994
	*S. litura*	GI	Xie et al., 1994
*T. Americana*	*S. litura*	GI	Wheeler et al., 2001
*Toona sp*.	*Oslrinia nubilalis*	GI	Arnason et al., 1987
*Trichilia havanensis*	*S. exigua*	FD,EI	Caballero et al., 2008
*Trichilia pallida*	*S. frugiperda*	FD	Bogorni and Vendramim, 2005
*T. pallens*	*S. frugiperda*	FD	Bogorni and Vendramim, 2005
*T. roka*	*S. frugiperda*	FD	Kubo and Klocke, 1982a,b
*Xylocarpus granatum*	*Mythimna separata*	FD	Wu et al., 2005

*EI, Enzyme Inhibition; GI, Growth Inhibition; FD, Feeding Deterrence; NPI, Nutritional Physiology Inhibition; OI, Oviposition Inhibition*.

**Figure 1:**
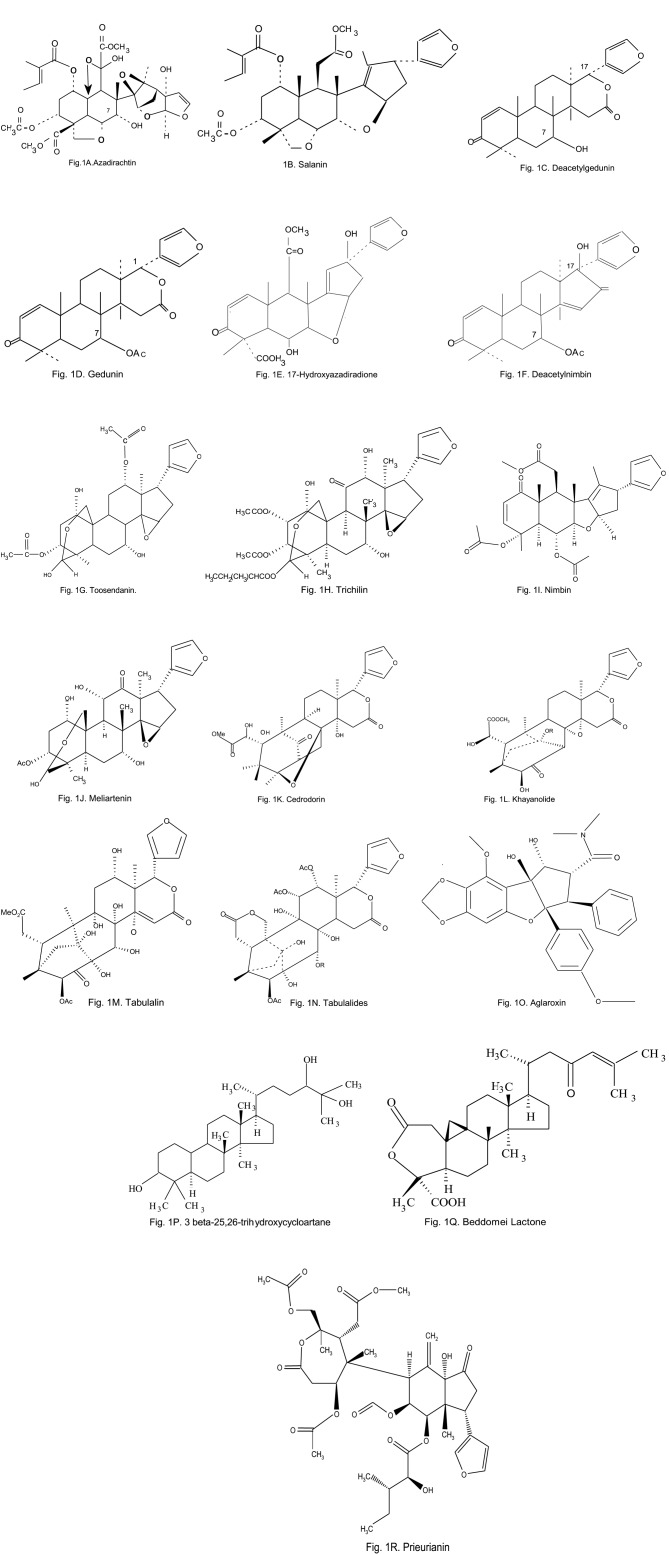
**Chemical structure of secondary metabolites identified from Meliaceae plants**.

Most work has focused on azadirachtin and other related compounds (Figures 1A–R) richly from neem seed extracts which act as both potent antifeedants and insect growth regulators. Azadirachtin and its content has antifeedent due to either hydrogenation of Δ^22^ double bonds or deacetylation caused any change by blocking of hydroxyl group affected the feeding inhibitory activity, while acetylation of azadirachtin caused a decrease in the activity maximum (Roy and Saraf, 2006). Further the stereo chemical structure around hemi acetyl region is important for antifeedent activity. Azadirachtin (Figure 1A) is a C-seco limonoid, which was isolated by Butterworth and Morgan (1968), as an insect feeding deterrent from the seeds of the Indian Neem tree, *A. indica* contain major limonoids, salannin, meliantriol, nimbin an other than azadirachtin. Azadirachtin affects the insect's reproductive organ, body development and other endocrine events (Mordue and Blackwell, 1993) and does not affect other biocontrol agent. Neem has affected more than 300 insect pests (Mordue and Blackwell, 1993). Further neem products are bio-degradable, mild toxic or no toxic to non-target organisms, while they are non-toxic toward humans and mammals (Mordue and Blackwell, 1993).

A closely relative of the neem tree is next to important for limonoids availability, *Melia azedarach* L. Extracts of the seeds are known to contain several limonoids and show excellent insecticidal activity (Srivastava, 1986; Lee et al., 1991; Charleston et al., 2005) but it has not affected biocontrol insects (i.e., predatory mite species (*Amblyseii* spp.), neem oil was also a feeding deterrent and toxin to Mythimna separata Walker. Apart from azadirachtin *M. azedarach* has the stem bark contain the limonoid toosendanin (Figure 1G) and this is the primary active ingredient of a botanical insecticide recently developed in China (Chiu, 1995). Toosendanin act as a feeding deterrent against Pieris rapae L (Chiu, 1989).

Further bioactive contents are available in few genera include *Cedrela, Khaya, Trichilia. Chisocheton Toona* and *Turaeu* (Isman, 1995; Abdelgaleil et al., 2001). *Aglaia* is another genus in the family Meliaceae and Aglaia was screened against *Peridroma saucia* Hubner. Seven species of *Aglaia* were possessed antifeedent activity against *P. saucia* of which were significantly inhibitory to growth (Satasook et al., 1992).

Koul et al. (2004) identified three major compounds, 3-O-acetyl salannol, salannol and salannin from *A. indica* (Figure 1B). All three compounds were found to affect nutritional indices of *Helicoverpa armigera* Hübner and *Spodoptera litura* Fab. Again Koul et al. (2005) found that *Aglaia elaeagnoidea* (Syn) was affected nutritional physiology of both *H. armigera* and *S. litura*. The compound aglaroxin A identified from *A. elaeagnoidea* was potent antifeedent against both Lepidopteran species (Figure 1O). The proved that the reduction in growth of the larvae was not entirely due to antifeedent, but partly due to the toxic effects of the aglaroxin A compound. Qi et al. (2003) have been identified compound munroniamide from *Munronia henryi* and that has proved antifeedent activity against *Pieris brassicae* L.

Besides the well-known antifeedant activity, azadirachtin also showed strong insect growth regulating activity against many insects (Schmutterer, 1990; Mordue and Blackwell, 1993). Since azadirachtin did not reduce feeding in *P. brassicae* pupae, the growth retardation and deformities were the direct effect of azadirachtin and not due to lack of food (Kraus and Grimminger, 1981). Nutritional analyses revealed that the insect growth inhibitory and antifeedant effects were independent of each other and relative to the level of treatment with (Ruscoe, 1972; Koul and Isman, 1991). Furthermore, 48 h feeding of on foliage treated at 5–10 ppm appeared to be sufficient for growth disruption of *S. litura* at early instars age (Kraus and Grimminger, 1981).

The insect growth regulating activity of azadirachtin focused its effects mainly on the molt of insects (Kraus and Grimminger, 1981). Feeding on azadirachtin-sprayed creeping bentgrass caused molting disorders and death of early instar *Agrotis ipsilon* and slowed feeding and stunted the growth of late instars (George and Potter, 2008) caused significant reduction in feeding activity at 2.5 g/L, prolonged the period for molting to nymphal stage, and caused 60% reduction in moltability. In addition, inhibited cold-induced supernumerary molt of last-instar *Galleria mellonella* and induced disturbances in larval and pupal ecdysis as well as in the metamorphic process, thus resulting in the formation of various intermediates (Malczewska et al., 1988; Al-Rajhy et al., 2003).

It seemed likely that pupation in azadirachtin-treated *Manduca sexta* was inhibited by a disturbed ecdysteroid regulation shortly before pupal ecdysis, and was able to inhibit development even when individuals performed a complete molt after the treatment (Schlüter et al., 1985). In preventing normal development of final-instar larvae of *Heliothis virescens*, apparently reduced molting hormone titers by reducing prothoracicotropic hormone (PTTH) titers and the receptivity of prothoracic glands to produce ecdysone via stimulation by PTTH. In *Mamestra brassicae* 3 ppm of azadirachtin caused degenerated spermatocysts (Shimizu, 1988). The morphological and biochemical effects induced by azadirachtin suggested a widespread blockade of factors presumably located in the central nervous system stimulated a specific deterrent neuron in the lepidopterous species tested and inhibited the firing of neurons with signal phagostimulants in another test (Rembold et al., 1984; Simmonds and Blaney, 1984).

The feeding experiments showed the ED_50_ values of sendanin (Burke et al., 1977) for growth inhibition against *Pectinophora gossypiella*, *Heliothis zea*, *H. virescens*, and *S. frugiperda* ranged from 9 to 60 ppm, with *P. gossypiella* being the most sensitive and *Heliothis* complex the least (Kubo and Klocke, 1982a,b). When incorporated into artificial diets of neonates at 50 ppm, humilinolides A-D (Niven and Taylor, 1988; Anderson and Ley, 1990; Anderson et al., 1991; Zhang et al., 2008a,b) caused larval mortality, as well as growth reduction and increased the development time of survivors in a concentration-dependent manner. In addition at 5 ppm also reduced growth and survivorship of *Ostrinia nubilalis*.(Jimenez et al., 1997a,b), Swietenin C (Zhang et al., 2008a,b), humilinolide E (Harrison et al., 1970), methyl-2-hydroxy- 3β-isobutyroxy-1-oxomeliac-8(30)-enate (Qi et al., 2004), and humilin B (Nicolaou et al., 2002) reduced survivorships at various stages against *Ostrinia nubilalis*, while 6α-acetoxygedunin (Akisanya et al., 1961) reduced growth at the test concentration of 50 ppm. (Jimenez et al., 1998), febrifugin A (Da Silva et al., 2008), the last showed the highest insecticidal activity at 50.0 mg/kg against *S. frugiperda*. Further 20, 21, 22, 23-tetrahydro-23-oxoazadirone (Kadota et al., 1990) showed insecticidal activity against *Peridroma saucia*.

The methanolic seed extract of *M. azedarach* treatment at 1% and 10% resulted in decrease in feeding was observed in a *S. frugiperda*. When increasing the concentrations of extract the larvae digested and/or metabolized the food with minimum level. The reduction in growth was not completely due to the starvation but also due to ingestion of toxic substances from *M. azedarach* (Breuer and Schmidt, 1995).

Macleod et al. (1990) found that the meliatoxins isolated from the ethanolic fruit extract from *M. azedarach* var. *australasica* was toxic on *S. litura* larvae. Further Meliatoxin significantly reduced the ingestion of food at 400 ppm (480 mg/cm^2^) and they pointed out that the C-15 keto group would be responsible for the growth inhibition. There are other genera in the Meliaceae that also contain limonoids that show promising pesticide activity. But much less work has been carried out on those species (De Sousa et al., 2009).

In insect alimentary canal midgut is generally considered as a tissue where the digestive enzymes secret and is a site for digestion and absorption of nutrients. Also it is an important tissue affected by many kinds of toxicants including entomopathogens (Sutherland et al., 2002a,b). Insect gut is differentiated in three regions that include foregut, midgut, and the hindgut. Further it signifies one of the most important areas in insect physiology because of interaction between the insects and the environment. Hence it has been the focus of entomologist aiming to develop effective methods of insect pest's control (Chapman, 1998; Levy et al., 2004). Among the three regions, the midgut region has particularly been the most studied, because alterations on it affect the growth and development of insects as a result of changes in the physiological events that depend on meal intake, absorption and transformation (Mordue and Blackwell, 1993; Nisbet, 2000; De Sousa et al., 2009). The epithelium of the midgut in Lepidoptera is composed of columnar cells which are responsible for absorption and enzymes secretion, goblet cells for ionic homeostasis, endocrine cells for endocrine function and the regenerative cells for epithelium renewal (Genta et al., 2006; Pinheiro et al., 2008; De Sousa et al., 2009).

The peritrophic membrane in the midgut is important cell organelle which has a fundamental role of protection of the midgut. The peritrophic membrane is located between the gut lumen and the epithelial layer. It is a protective layer, protecting this epithelium from mechanical damage and it protect against toxic materials to the insect (Terra, 2001). Lot of works has been done on the morphological and ultrastructure of insects midgut from Lepidoptera such as *Diatraea saccharalis* (Fabricius), *Manduca sexta* L., *Spodoptera frugiperda*, *Anticarsia gemmatalis* (Hübner), *Alabama argillacea* (Hübner) suggest that the distribution and morphology of the epithelial cells can vary along this region (Pinheiro et al., 2003, 2008; Levy et al., 2004; De Sousa et al., 2009). These differences are usually observed at the ultrastructural level (Santos et al., 1984; Billingsley and Lehane, 1996).

Insect midgut cells synthesizing and secreting digestive enzymes. These enzymes can be divided into two types. One is constitutively secreting cells and they do not accumulate secretory products. Also synthesized enzymes may release immediately after their synthesis. Regulated secretory cells collect secretory material which is quickly released in response to a suitable signal (Lehane et al., 1995). Also during the digestion process ingested macromolecules are break down into smaller parts by the insects and it will be absorbed by the epithelial cells in midgut. Further many enzymes has play vital role during this process. During the digestive cycle, there are significant changes in the levels of midgut digestive enzymes. This suggests that digestive enzyme synthesis and secretion are controlled during the digestive process (Lehane et al., 1995).

There is four categories of control mechanism of digestive enzyme levels in insects have been identified so for. That is included as-nervous, hormonal, paracrine and prandial. Direct nervous control of digestive enzyme synthesis has been largely discounted on the grounds that innervation appears limited to motor innervation of the midgut musculature (Day and Powning, 1949; Garcia and Garcia, 1977; Žitòan et al., 1993; Lehane et al., 1995). The pH of gut contents is one of the most important factors that affect digestive enzymes. Many determinations have been reported so for about the luminal pH values in many insects with pH optima of their digestive enzymes. These studies headed to the claim that there is a correlation between enzyme pH optima and luminal pH in insect guts (Applebaum, 1985; Terra and Ferreira, 1994). First, most of the pH data's were obtained by measuring contents of entire midguts, thus mixing contents of different midgut regions including foregut, midgut and hindgut which are now known to have contrasting pH values in several insects (Terra and Ferreira, 1994). Lepidopteran insects may display varying pH alkaline contents, particularly in the middle ventriculus, as they are herbivorous (eat leaves), wax (*Galleria mellonella*) or keratin (*Tineola bisselliella*). This high pH may be an adaptation of leaf-eating Lepidopteran families for extracting hemicelluloses from plant cell walls (Ferreira et al., 1988; Terra and Ferreira, 1994). The pH of the midgut is usually in the range 6–7.5. The higher alkalinity of the midgut contents (pH 9–12) was already described in Lepidopteran (Houseman and Downe, 1980; Terra, 1990).

Digestive enzymes are hydrolases. Enzymes liable for the hydrolysis of proteins down to amino acids are the proteases. Proteases (peptide hydrolases, EC 3.4) are enzymes acting on peptide bonds and include the proteinases (endopeptidases, EC 3.4.21-24) and the exopeptidases (EC 3.2.4.11-19). Proteinases are divided into sub-classes on the origin of catalytic mechanism (Terra and Ferreira, 1994; Lehane et al., 1995; Terra et al., 1996; Shekari et al., 2008). Trypsins (EC 3.4.21.4) are serine proteinases that will cleave protein chains on the carboxyl side of basic L- amino acids. The enzyme is exactly inhibited by N-α-tosyl-L-lysine chloromethyl keton which acts on histidine (Shaw et al., 1965; Terra and Ferreira, 1994). Apart from this Chymotrypsins (EC 3.4.21.1), cathepsin B (EC 3.4.22.1.), pepsin (EC 3.4.23.1), Aminopeptidases (EC 3.4.11.), Carboxypeptidases (EC 3.4.16-18) and Dipeptidases (EC 3.4.13.) are major proteases digestive enzymes.

Carbohydrase is responsible for catalyzes the breakdown of carbohydrates into simple sugars. It includes α-Amylase (EC 3.2.1.1), β-amylase (EC 3.2.1.2), glucoamylase (EC 3.2.1.3), exo-β-l,4-glucanases (EC 3.2.1.91), endo-β-l,4-glucanases (EC 3.2.1.4), β-l,4-glucosidases (EC 3.2.1.21), chitinase (EC 3.2.1.14), β-Nacetyl-D-glucosaminidase (EC 3.2.1.52), Lysozyme (EC 3.2.1.17), Lysozyme (EC 3.2.1.17), α-Glucosidases (EC 3.2.1.20), and Trehalase (EC 3.2.1.28) (Wyatt, 1967; Huber and Mathison, 1976; Applebaum, 1985; Dunn, 1986; Kramer and Koga, 1986; Martin et al., 1991). Further Christeller et al. (1992) identified midgut protease activities in midgut was higher in Lepidopteran insects from the families, Tortricidae, Noctuidae, Gelechiidae, Hepialidae and Pyralidae. Further treatment with chemical insecticides has directly affected the digestive enzyme including amylase, invertase, lipase, and protease (Deshmukh et al., 2009).

Alkaline phosphatase (ALP, E.C.3.1.3.1) and acid phosphatase (ACP, E.C.3.1.3.2) are hydrolytic enzymes, which hydrolyse phosphomonoesters under acid or alkaline conditions, respectively. ALP is mainly found in the intestinal epithelium of animals and its primary function is to provide phosphate ions from mononucleotide and ribonucleo-proteins for a variety of metabolic processes. ALP is involved in the transphosphorylation reaction (Sakharov et al., 1989). Adenosine triphosphatases (ATPases) are essential for the transport of glucose, amino acids, and other organic molecules. Any impairment in their activity will affect the physiology of the insect gut. These enzymes are located in the midgut, malpighian tubules, muscles, and nerve fibers of the Lepidopertan insects (Horie, 1958). Midgut has the highest ALP and ACP activity as compared to other tissues. The ALP and ACP activities are low during the larval moulting stage and increased gradually after moulting (Miao, 2002). The highest activity appeared before the full appetite gluttonous stage fifth instar and the lowest activity was found in the mature larval stage (Miao, 2002; Senthil-Nathan et al., 2005a,b,c,d).

Lactate dehydrogenase (LDH) (EC 1.1.1.28) is an important glycolytic enzyme present in virtually all animal tissues (Kaplan and Pesce, 1996). It is also involved in carbohydrate metabolism and has been used to indicate exposure to chemical stress (Wu and Lam, 1997; Diamantino et al., 2001). LDH is a parameter widely used in toxicology and in clinical chemistry to diagnose cell, tissue and organ damage. However, the potential of this enzyme as an indicative criterion in invertebrate toxicity tests has been scarcely explored (Ribeiro et al., 1999).

## Effect of meliaceae secondary metabolites on nutritional indices

Nutritional/food utilization efficiencies of insects characteristically calculated and expressed as percentages of approximate digestibility (AD) or assimilation efficiency (AE- absorption or digestive efficiency) estimates the percentage of ingested food that is digested and assimilated. Efficiency of conversion of digested food (ECD) or net growth efficiency (NGE; sometimes metabolic efficiency) estimates the percentage of assimilated food that is converted to biomass; and efficiency of conversion (to biomass) of ingested food (ECI) or gross growth efficiency (GGE- growth efficiency) estimates the percentage of ingested food that is converted to biomass (Waldbauer, 1968; Slansky, 1985; Slansky and Scriber, 1985).

Incorporation of azadirachtin, salannin, and nimbinene limonoids from neem into the artificial diet of fourth instar larvae significantly reduced the consumption and relative growth of *S. litura* larvae compared to controls at 4, 8, and 1.2 ppm concentrations tested. But Efficiency of conversion of ingested and digested food (ECI and ECD) into biomass of *S. litura* larvae was not reduced. Approximate digestibility (AD) was continued to be same in all treatments. Interestingly, both ECI and ECD were reduced at all doses after topical application 0.1, 0.5, and 1 μg/Ins of azadirachtin to fourth instar larvae with a considerable decrease in relative growth rate. The reduction in the food utilization experiment was regardless of any significant change in relative consumption rate (Koul et al., 1996).

Treatment with aglaroxin A (Figure 1O) (1, 3, and 5ppm) from *Aglaia elaeagnoidea* caused reduced RGR and RCR with a significant change in the ECI values on both *H. armigera* and *S. litura*. Reduction in growth was not only correlated with dietary concentrations. When the compounds were applied topically to the 3rd instar larvae, significantly affect the larval growth and ECI parameters but the consumption was not reduced significantly (Koul et al., 2005). Further Koul et al. (2005) confirmed physiological toxicity of aglaroxin A by comparing of RGR and RCR values. They proved the reduced growth of these larvae under the effect of aglaroxin A was not completely due to starvation; some of the growth reduction was due to the toxic effect of aglaroxin A.

Further Wheeler and Isman (2001) described 25, 50, 75, 100, and 250 ppm of dietary concentration and 2.5, 5.0, and 10 topical applied doses (μg insect^−1^). Nutritional analyses revealed that the extract also acts as a chronic toxin when ingested by larvae. The crude extract, when incorporated into artificial diet reduced RGR, RCR, ECI, and ECD in a dose dependent manner.

**Figure 2:**
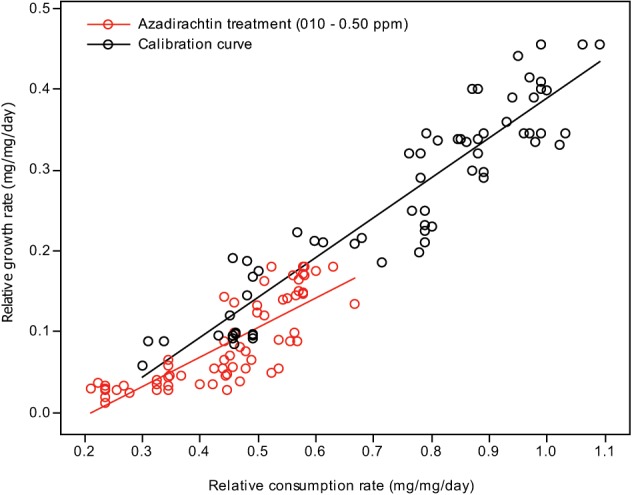
**Correlation between the relative consumption rates and relative growth rates of *C. medinalis* fed on different quantities of control diet (calibration curve) and larvae fed on diet containing different concentrations of azadirachtin**.

**Figure 3:**
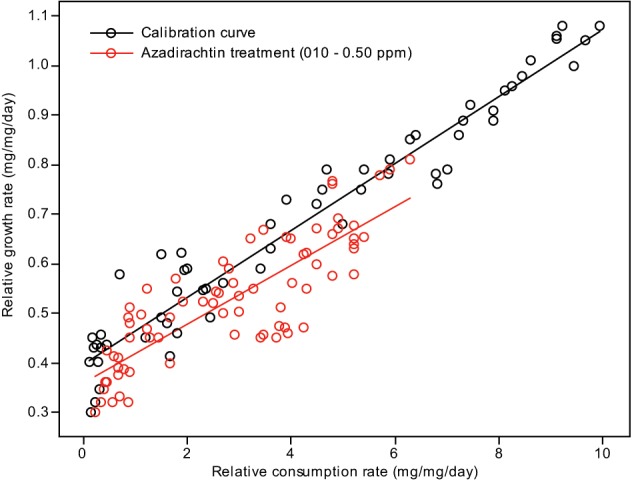
**Correlation between the relative consumption rates and relative growth rates of *S. litura* fed on different quantities of control diet (calibration curve) and larvae fed on diet containing different concentrations of azadirachtin**.

Plotting relative growth rates against consumption rates was used to estimate the differentiation between the treatment doses and control in toxicological assay. Two lines were generated for each: one calibration curve, where a range of RCRs were generated and correlated to the RGRs, and one test line, where the larvae were fed diets containing different treatment doses of compound concentrations. The RGR and RCR for each set of larvae were subjected to a linear regression analysis (Figures 2, 3). The slope (regression coefficient) of the regression line represents the growth efficiency of the larvae. The two regression coefficients were compared by calculating the variance of the difference between the two estimates of the regression coefficients (Anderson et al., 1977; Searle, 1977; Wheeler and Isman, 2001; Koul et al., 2005; Senthil-Nathan et al., 2009; Chandrasekaran et al., 2012). This test showed that the growth efficiency of *Cnaphalocrocis medinalis* Guenée and *S. litura* fed on a treated diet was significantly less than that of the control larvae with the insects fed in three different concentrations growing differently for a given RCR. This again indicates that the reduced growth of these larvae under the influence of azadirachtin is not entirely due to starvation; some of the growth reduction is due to toxic effect of the pure limonoids azadirachtin.

Barnby and Klocke (1987) have reported neem feeding inhibition to a direct action of azadirachtin on the “centers that control feeding and metabolism.” Although azadirachtin treatment decreased food intake by *S. littoralis* larvae, this reduction alone would not explain the pronounced inability of the larvae to gain weight in the instars immediately after treatment. Reductions in weight gain were also observed in the sixth instar, but accompanied by no reduction in food intake in *S. litura* and (Ayyangar and Rao, 1989; Ramachandran et al., 1989) and *S. exempta* (Tanzubil and McCaffery, 1990).

Wheeler and Slansky (1991) and Slansky (1993) described that digestibility may not be closely connected with retention time of food in the gut. Adverse effects of azadirachtin on midgut epithelial cells, which might disrupt enzyme secretion and nutrient absorption, have been reported (Nasiruddin and Mordue Luntz, 1993).

Timmins and Reynolds (1992) pointed out a reduction in the efficiency of food utilization following *M. sexta* treatment with azadirachtin to increased energetic costs arising from a reduced ability to utilize dietary nitrogen, which would not necessarily interfere with absorption from the gut (digestibility). They further pointed out that, in the absence of an essential supply of minerals, amino acids and other nutrients then in excess for growth might be diverted into other metabolic pathways. Many of researchers have further proposed that such other pathways might include those involved in detoxification of allelochemicals like limonoids (Arnason et al., 1985; Barnby and Klocke, 1987; Tanzubil and McCaffery, 1990; Martinez and Van Emden, 1999; Senthil-Nathan et al., 2005d, 2007; Senthil-Nathan, 2006).

Experiments with azadirachtin on *C. medinalis* and *S. litura* were carried out to investigate whether the efficacy was purely a feeding deterrence or toxicity mediated physiological inhibition (Senthil-Nathan, unpublished data). Using food utilization measurement, it was established that there was a reduction in growth rate associate with the decrease in consumption, which accounted partially for the decrease in growth rate as there was a reduction in ECI values. ECI is a complete measure of an insect's capacity to utilize the food that it ingests for growth. Therefore, a change in ECI values indicates that ingested secondary metabolites exhibit toxicity, and is not just an antifeedent affect (Koul et al., 2005).

Reduced RGR and RCR was also observed after treatment with *M. azedarach* on *S. frugiperda* (Breuer and Schmidt, 1996) and *C. medinalis* (Senthil-Nathan, 2006a,b,c).

## Effect of meliaceae secondary metabolites on digestive enzymatic profiles of Lepidoptera

The effects of neem derivatives azadirachtin on the fourth instar larvae of *Plodia interpunctella* Guenée, resulted in severe reduction in protein, glycogen and lipid contents 7 days after treatment. Further the α-amylase activity on polyacrylamide gel showed a weak enzymatic activity in larvae fed azadirachtin indicating a severe reduction in a-amylase activity (Rharrabe et al., 2008). Further treatment with azadirachtin directly/indirectly inhibits the production of trypsin by the enzyme-secreting cells of the midgut wall of *M. sexta* (Timmins and Reynolds, 1992). Also Timmins and Reynolds (1992) suggest that inhibition of either synthesis or release of trypsin due to azadirachtin might be a direct action on the enzyme-secreting cells of the midgut wall. Azadirachtin may act indirectly, by disturbing some mechanism that might control trypsin secretion. Most of the Lepidopteran insect, possess endocrine cells associated with the midgut wall (Endo and Nishiitsutsuji-Uwo, 1980). The endocrine cells may responsible for local control of enzyme secretion into the gut lumen. Further circulating hormones from the classical neuroendocrine system might act to control enzyme levels. These are all preliminary finding but it is well-known that known that azadirachtin may affect the secretory function of neuroendocrine cells in insects (Barnby and Klocke, 1990; Garcia et al., 1990). Rharrabe et al. (2008) observed that exposure to azadirachtin, a significant decrease in protein, glycogen and lipid contents was observed in *P. interpunctella* Hübner. The reduction of such biochemical contents can be due to major mobilization of these substances in reaction to the absence of nutrients caused by the toxic effect of azadirachtin on the midgut and a decrease of their synthesis. The walls and epithelial cell of the digestive tract in insects have a high content of detoxification enzymes, as a barrier to plant secondary metabolites hat they may consume with the diet (Ortego et al., 1999).

Hasheminia et al. (2011) has clearly pointed out that treatment with plant extract to Lepidopteran insect hinder the link between the carbohydrates and protein metabolism and are altered during various physiological processes aminotransferases. Further they stated that plant extracts exhibited an endocrine disruption by way of progressive or retrogressive larval duration, this explanation could be pointed out for reduced alanine aminotrasferase (ALT) and aspartate aminotransferase (AST). Smirle et al. (1996) stated that changes in metabolism and decreases in the protein content of neem-treated individuals may be expected to affect enzyme titers of *Choristoneura rosaceana* L. especially glutathione S-transferases.

Senthil-Nathan et al. (2004) observed that changes in acid phosphatases (ACP), alkaline phosphatases (ALP) and adenosine triphosphatases (ATPase) activities after treatment with neem extracts in *C. medinalis*. They concluded that changing the physiological balance of the midgut might affect the enzyme activity. ALP is involved in the transphosphorylation reaction. In their study, the decrease in the activity of these enzymes after treatment with neem extract suggests that these materials affect gut physiological events (i.e., ion transport) that might influence these enzymes (Phillips et al., 1988). Decreased level of ACP at higher concentration of neem extract suggests reduced phosphorus liberation for energy metabolism, decreased rate of metabolism, as well as decreased rate of transport of metabolites, and may be due to the direct effect of neem seed extract on *C. medinalis* (Senthil-Nathan et al., 2004, 2006d,e).

**Figure 4:**
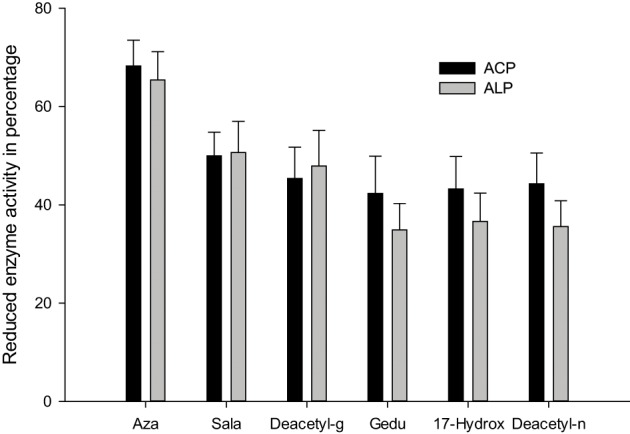
**Activity of ACP and ALP against the 1 ppm treatment of azadirachtin on *C. medinalis***.

ATPases are essential for transport of glucose, amino acids, etc. Any impairment in their activity will affect the physiology of the gut. The role of membrane lipids and their micro-environmental changes at the physical and chemical levels may be responsible for the differential response observed at the level of ATPase activity after treatment with neem extract against the *C. medinalis*. Membrane ATPase, especially in the intestinal epithelium, assists transport and reabsorption of metabolites and nutrients and also secondary transport of ions and non-electrolytes (Lechleitner and Phillips, 1988; Fogg et al., 1991). Babu et al. (1996) showed that the ATPase activity in the gut of *H. armigera* was significantly decreased, due to toxic effects of azadirachtin. ATPase inhibition may affect active ion transport, leading to alteration in electrolyte regulation. After neem extract treatment a decrease in enzymatic activity denotes reduced metabolism in the insect and may be due to the toxic effects neem compounds on membrane permeability, especially on the gut epithelium (Figure 4) (Senthil-Nathan et al., 2005a,b, 2007).

Meliaceous plant compounds indicate that there may be effects on enzyme titers and activities (Klocke, 1989; Feng et al., 1995). Feeding is necessary for the stimulation of digestive enzyme activities (Smirle et al., 1996; Shekari et al., 2008) and may have interfered with the enzyme–substrate complex thus affecting the peristaltic movement of the gut (Broadway and Duffey, 1988; Duffey and Stout, 1996) a phenomenon that was very clear observed by the decrease of fecal pellet production in the *M. azedarach* treatment (Senthil-Nathan, 2006).

**Figure 5:**
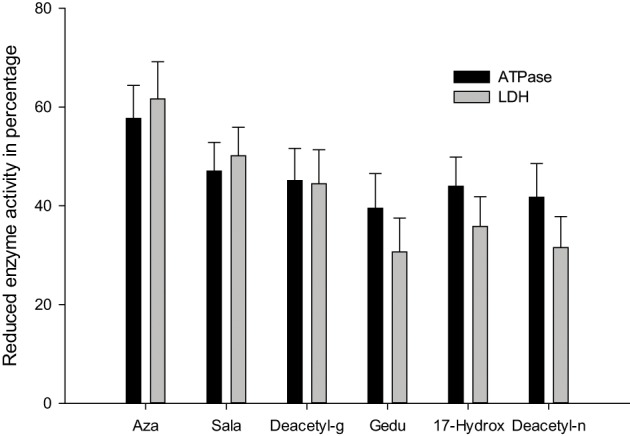
**Activity of ATPase and LDH against the 1 ppm treatment of azadirachtin on *C. medinalis***.

**Figure 6:**
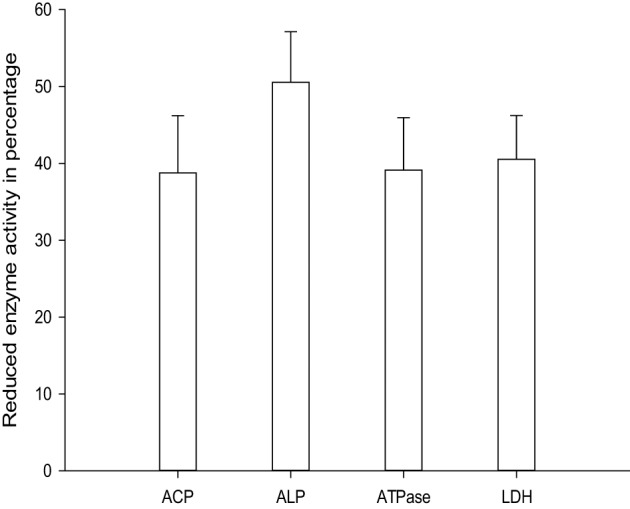
**Midgut enzyme activity of *S. litura* after treatment with 1 ppm azadirachtin**.

**Figure 7:**
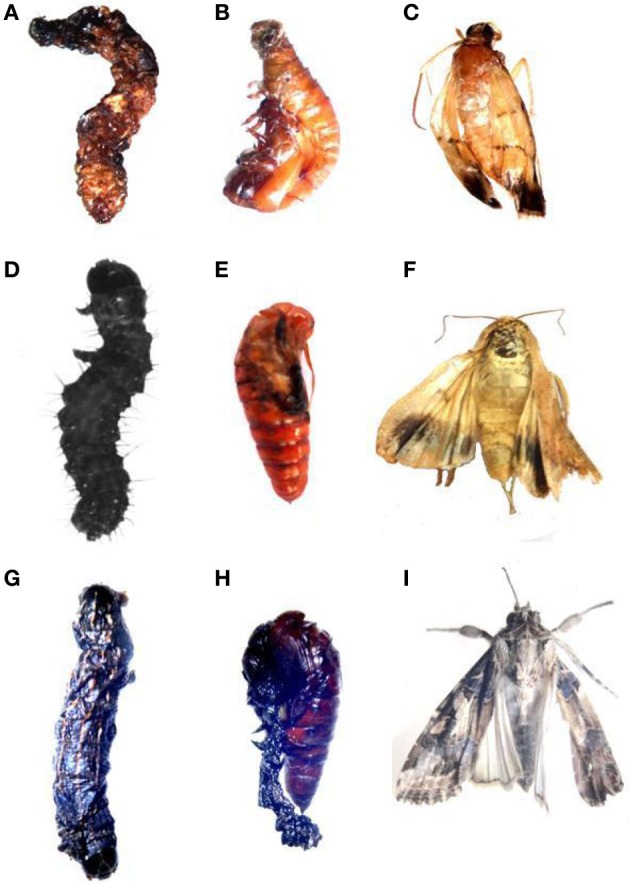
**Larval deformities of Lepidopteran insects after treatment with 0.5 ppm of Azadirachtin. (A–C)** *C. medinalis* larval, pupal and adult deformities. **(D–F)** *H. armigera* larval, pupal and adult deformities. **(G–I)** *S. litura* larval, pupal and adult deformities.

Lactate dehydrogenase (LDH) (EC 1.1.1.27) is involved in the production of energy, being particularly important when a considerable amount of additional energy is required immediately. A negative correlation between LDH activity and ambient oxygen levels for some aquatic organisms were suggesting a possible biochemical adjustment in response to the lowered oxygen levels. This probably occurs also in situations of chemical stress. Therefore, this enzyme may be a sensitive criterion in laboratory (Zebe and McShan, 1957). After treatment with neem limonoids a decrease in LDH activity denotes reduced metabolism in the insect and may be due to the toxic effects of neem derivatives on membrane permeability, especially of the gut epithelium (Figures 5–7) (Senthil-Nathan et al., 2005b, 2006a,b,c,d,e; Zibaee et al., 2008). Further Mitchell et al. (1997) identified neem compounds inhibit ecdysone 20-monooxygenase activity associated with fat body and midgut of fifth instar larvae of *M. sexta*.

## Effect of meliaceae secondary metabolites on NADPH cytochromec reductase and cholinesterase

Artificial diet containing 0.01% of an ethyl acetate fraction of *M. azedarach* fruit extract inhibited the cholinesterase activity of the larvae of *S. frugiperda* (Breuer et al., 2003). It is known that this detoxification system becomes more Role of *M. azedarach* L. (Meliaceae) for the control of insects activated as larvae develop (Breuer et al., 2003), which would explain the lower sensitivity to treatments of the bigger larvae (Breuer and Schmidt, 1996; Yasmin et al., 2010).

This increase suggests that the cytochrome-P-450-system might be involved in the detoxification mechanism, because this enzyme is the most important flavoprotein component within the microsomal electron transfer chain. Cytochrome-P-450 enzymes are known to degrade various substrates, especially lipophilic ones (toxicants) and are involved in the elimination of insecticides. The capacity to inactivate natural compounds, such as flavenoids and terpenoids, has also been demonstrated before (Brattsten et al., 1977; Dowd et al., 1983; Yu, 1983). Similar components are present in *M. azedarach* (Kraus, 1986; Breuer et al., 2003). Bullangpoti et al. (2012) proved that *in vitro* experiments with *M. azedarach* senescent leaf extracts inhibit esterases and P450 enzymes. Also Feng et al. (1995) clearly pointed out the extract of *M. toosendan* inhibit midgut esterases of *S. litura*

This review indicates that there is a possible interaction between Meliaceae secondary metabolites and gut enzymes. Meliaceae limonoids like azadirachtin may directly influence the expression of this receptor (Huang et al., 2004) it could cause a major disruption to the growth, and development of an insect. Further it could make Meliaceae secondary metabolites an important tool in the management of resistant populations of Lepidopteran where enzyme based metabolism is involved.

### Conflict of interest statement

The author declares that the research was conducted in the absence of any commercial or financial relationships that could be construed as a potential conflict of interest.
